# Editorial: Inflammation and Fibrosis in the Gastrointestinal Tract and Liver: Mechanisms and Targets

**DOI:** 10.3389/fphar.2021.773228

**Published:** 2021-09-24

**Authors:** Ralf Weiskirchen, Dario Sorrentino, Wolfgang R. Stremmel

**Affiliations:** ^1^ Institute for Molecular Pathobiochemistry, Experimental Gene Therapy and Clinical Chemistry (IFMPEGKC), RWTH University Hospital Aachen, Aachen, Germany; ^2^ IBD Center, Division of Gastroenterology, Virginia Tech Carilion School of Medicine, Roanoke, VA, United States; ^3^ Practice for Internal Medicine, Baden-Baden, Germany

**Keywords:** liver, gastrointestinal tract, IBD, inflammation, fibrosis, therapy

## Introduction

Inflammation of the gastrointestinal tract and liver can be caused by a variety of factors including genetic diseases, immune mediated conditions, environmentally induced reactions, toxic exposure, tissue injury, abnormalities in the gut-liver axis, and general infectious diseases. The consequence of inflammation is wound healing following fibrous tissue repair and regeneration. Although it occurs in all organs, each organ shows a specific pattern of reactivity.

In this Research Topic, leading experts from the field of Gastroenterology and Hepatology share new findings and current concepts in the pathogenesis and management of inflammation and fibrosis in the gastrointestinal tract and liver. In total about 200 basic scientists and clinicians from eight countries (China, Egypt, Germany, India, Japan, Spain, Italy, and USA) contributed 28 originals or review articles about current perspectives and findings in corresponding diseases. These articles cover a wide range of aspects in the pathogenesis of liver inflammation and fibrosis, gastrointestinal disease, and inflammatory bowel disease (IBD). The contributions show that these fascinating fields have made conceptual advances that in the near future will hopefully lead to novel treatment options.

## Liver Inflammation and Fibrosis

An et al. discuss the main roles of interleukins (ILs) in the context of hepatic inflammation and how this knowledge can be used for the development of therapeutic drugs. In particular, they highlight the dynamics by which interleukins mediate the cross-talk between hepatic cells during liver inflammation and injury. It becomes clear that abrogating IL signaling might prevent the progression of hepatic fibrogenesis in the early stages of liver injury. However, as the authors discuss there are some practical problems that need to be addressed to move IL-targeted therapies from the experimental stage to the clinical setting.

In an experimental animal study, Han et al. investigated the impact of a traditional medicinal herbal extract derived from Chicory (*Cichorium pumilum Jacq*) in a rat model of liver fibrosis induced by chronic colitis. The authors demonstrate that this herb can promote probiotic growth and prevent liver fibrosis. In addition, the authors demonstrate that Lactucin that is one of the bitter tasting sesquiterpene lactone of Chicory is able to inhibit lipopolysaccharide-induced inflammatory responses *in vitro* and activation of the Mitogen-activated protein kinase and Akt signaling. Therefore, the authors proposed Lactucin as one of the most significant anti-inflammatory compounds in Chicory extracts.

Another compound boosting endogenous antioxidant mechanisms in the context of acetaminophen (APAP)-induced liver damage was identified by Rahman et al. In their study, the authors provide evidence that carveol improves liver detriments and liver metabolic deficits in mice subjected to APAP, while the nuclear factor erythroid 2-related factor 2 (Nrf2) inhibitor all-trans retinoic acid exaggerated APAP toxicity. Mechanistically, the authors found that carveol treatment increases *Nrf2* expression that combats various reactive oxygen species and other stress kinases. Additional, comprehensible *in-silico* docking studies showed that carveol might have an affinity to the active catalytic pockets of COX-2, HO-1, IL-1, NF-κB, iNOS, Nrf2 and TNF-α that all impact inflammation.

The *Nrf2* pathway was also investigated in an experiment study by Zhao et al., who analyzed the effect of bicyclol in cholestatic mice caused by bile duct ligation. Interestingly, it was found that bicyclol acts as a significant hepatoprotective agent that attenuates liver damage by increasing the levels of hydropholic bile acids such as α-muricholic acid (MCA) and β-MCA. Moreover, bicyclol promoted autophagy and activated *Nrf2* expression and antioxidant downstream genes.

Pu et al. provide evidence that the 5-lipoxygenase (5-LO) that catalyzes the oxidation of essential fatty acid substrates into inflammatory leukotrienes is a key enzyme involved in mediating hepatic fibrosis. The authors could show that genetic ablation or pharmacological inhibition of 5-LO is therapeutically beneficial to block hepatic fibrosis *in vitro* and *in vivo*. In line, patients suffering from non-alcoholic steatohepatitis and liver fibrosis showed elevated levels of 5-LO suggesting that strategies to target 5-LO may offer new therapeutic avenues.

A new network in the pathogenesis of hepatic fibrosis was introduced by Shouman et al. They evaluated antisense oligonucleotides directed against the tissue factor in rats in which liver fibrosis were induced by a single administration of Diethylnitrosamine followed by repeated doses of carbon tetrachloride once weekly for 6 weeks. The authors could demonstrate that the blockage of tissue factor expression resulted in a significant downregulation of the protease-activated receptor 1 (PAR1) and toll-like receptor 4 (TLR4) that are both critical drivers in hepatic inflammation and fibrosis.

Roeb and Weiskirchen summarized findings from a selective literature search on topics related to non-alcoholic fatty liver disease (NAFLD), fructose and fibrosis. The study showed that the rate of overweight and obesity is significantly higher in adult and pediatric patients suffering from non-alcoholic steatohepatitis (NASH). Although it is presently not known whether this is due to an excess of energy or the particular metabolism of fructose, the authors suggest that reduction in sugar consumption in conjunction with avoidance of foods enriched in saturated fats and weight loss is recommended for NAFLD and NASH patients.

Xiang et al. analyzed the effects of kaempferol on the liver X receptor α (LXRα)/lysophosphatidylcholine acyltransferase 3 (LPCAT3) pathway in livers of mice fed a high fat diet inducing NASH. They could show that this natural flavonol reduces endoplasmic reticulum stress and inflammation as assessed *in vitro* and *in vivo* by reduced expression of LXRα, LPCAT3 and genes involved in the initiation or execution of endoplasmic reticulum stress. Based on their results the authors conclude that the LXRα/LPCAT network offers new potential targets for NASH treatment.

Acharya et al. summarized recent aspects in the understanding of hepatic fibrosis including relevant disease-associated pathways, cellular and molecular drivers of fibrogenesis, and how this knowledge can be translated into therapy of respective disease. The review shows that hepatic fibrogenesis is a highly complex and orchestrated process that offers many therapeutic opportunities for impeding or reversing this disease process.

The sodium-glucose cotransporter two inhibitor dapaglifloxin was shown by Li et al. ameliorates hepatic steatosis by decreasing the *de novo* lipogenesis enzyme acetyl-CoA carboxylase 1 (ACC1), increasing the fatty oxidation enzyme acyl-CoA oxidase 1 (ACOX1), and inducing autophagy via the AMPK-mTOR pathway. This may be in future used as therapeutic approach to treat NAFLD by a functional mechanism.

The group of Ye et al. showed that Salidroside inhibits carbon tetrachloride-induced liver fibrosis in mice by suppression of JNK activation and modulating the sphingosine kinase 1/sphingosine-1-phosphate signaling pathway for inhibition of Akt. This reduces activation and migration of hepatic stellate cells (HSC) as the executive players in fibrosis. Salidroside alleviates by this mechanism liver injury, hepatocyte apoptosis and consequently liver fibrosis.

A review article by Drescher et al. discusses developmental differences between individual immune cells and their role in health and disease, with a focus on liver diseases. They summarized findings showing there exists a fragile balance between T helper 17 (T_H_17) and regulatory T cells (T_Reg_) that is critical in maintaining immune homeostasis during acute and chronic inflammation.

Zhu et al. focused on Hengshun aromatic vinegar as a food additive to improve NAFLD. It improved cell viability and attenuated cell damage. Serum levels of triglycerides, transaminases (ALT, AST) and malondialdehyde (MDA) dropped. Inflammation and lipogenesis were reduced by interference with metabolic key regulators, i.e. by enhancing silent information regulator of transcription 1 (Sirt1). Thus, it represents a new assistant strategy to fight NAFLD.

Kong et al. investigated the role of the AMP analog 5-aminoimidazole-4-carboxamide (AICAR) which acts as an activator of the AMP-kinase (AMPK) on the pathogenesis of acute pancreatitis-associated liver injury (PALI). In a rat model of sodium taurocholate-induced acute pancreatitis, they found that AICAR attenuated PALI and restored liver function. Moreover, the compound activated the AMPK/Nrf2 signaling pathway, while the AMPK inhibitor Compound C aggravated PALI. In Nrf2 null mice, the effect of AICAR on alleviation of liver injury was significantly weakened suggesting that AICAR protects against PALI by interfering with *Nrf2*-triggered processes.

## Gastrointestinal Tract

Lamas-Paz et al. identified a novel mechanism that triggers acute alcoholic hepatic injury in the context of the gut-liver axis. The authors could demonstrate mice subjected to acute alcohol injury develop significant alterations in the gut microbiota and in intestinal epithelial barrier function. Interestingly, these alterations are triggered by yet unknown mediators released from extracellular vesicles produced by intestinal epithelial cells provoking deleterious effects on hepatocyte viability and steatosis.

A thematically related study by Ren et al. demonstrated that the COX-2 inhibitor rutaecaprine has gastroprotective effects on ethanol-induced acute gastric mucosal injury in mice. In the respective model, rutaecaprine augmented cellular antioxidant capacity, most likely by triggering antioxidant and anti-apoptosis defense capacities that results in inhibition of the inflammatory acting NF-κB, pathway.

Chen et al. performed an ultra-high performance liquid chromatography-quadrupole time-of-flight mass spectrometry (UPLC-Q-TOF/MS)-based study to investigate the ameliorative effects of palmatine on *Heliobacter pylori*-induced chronic atrophic gastritis in rats using serum and urine metabolomics. Interestingly, this alkaloid attenuated pathological damage and inflammation and improved integrity of the gastric mucosal epithelial barrier. Mechanistically, the authors suggested that the therapeutic effects of palmatine are majorly attributable to its capacity to impact the metabolism of taurine, subtaurine, glycerol phospholipid and the mutual transformation of pentose and glucoronide.

Yang et al. found that Gremlin 1 (GREM1) is significantly increased in mouse fibrotic colon. In their original study, the authors demonstrated that GREM1 promoted the proliferations and activation of intestinal fibroblasts by activating the VEGFR2 receptor and triggering fatty acid oxidation. In line with these findings, the authors could block intestinal fibrosis progression *in vivo* by blocking the GREM1-VEGFR2 axis.

## Inflammatory Bowel Disease

Enck and Klosterhalfen addressed the interesting question why in trials of irritable bowel syndrome (IBS) and IBD the placebo rates are remarkably high. Based on a meta-analysis, they report that the spontaneous improvement accounts for 50% of the placebo effect. Moreover, nocebo effects are easily detectable in randomized control trials (RCTs), whereas adverse events are difficult to see in respective reports.

Chen Y et al. developed a novel prediction model useful to estimate the risk of primary non-response rate to infliximab therapy and select the optimal treatment for individual patients suffering from Crohn’s disease. In their retrospective study including 322 Crohn’s disease patients and data from the Gene Expression Omnibus (GEO) database, the authors performed and intensive bioinformatics analysis and provide evidence that IL-6 is a good predictive factor for risk assessment.

Schmidt et al. evaluated the clinical efficacy of tumor necrosis factor antibodies on chronic IBD outcome. In total one third of the patients do not respond and in many cases drug efficacy is lost over time. Contraindications, adverse events and intolerance have to be considered when these biologicals are administered. This overview of new therapeutic approaches with novel biologicals is helpful for clinicians treating IBD.

The group of Zhong et al. showed that the serine-threonine kinase inhibitor rapamycin is effective in the treatment of upper gastrointestinal Crohn’s disease-related stricture formation, but has no effect in lower gastrointestinal Crohn’s disease. The drug was recently shown to reduce intestinal fibrosis by inhibiting CX3Cr1-mTOR-induced autophagy in mononuclear phagocytes and upregulating the IL-23/IL-22 axis. This was now evaluated by a Gastrointestinal obstruction symptoms score and diet score, which both showed improvement in upper gastrointestinal Crohn’s disease, but not in lower gut stricture disease. Adverse events were recorded in 40% of the patients, mostly mouth ulcers. However, no death or serious opportunistic infections were observed. This therapy may avoid surgical or endoscopic interventions. These promising results should now be confirmed in randomized trials with larger numbers of patients.

Al-Bawardy et al. highlighted the relevance of continued development of new therapeutic strategies and modifications of existing therapies to improve the outcome of IBD. In particular, they suggested small molecule inhibitors targeting the Janus kinase or the sphingosine-1-phosphate receptor that both critically contribute to the pathogenesis of IBD as promising therapeutic targets. Most important is that most of these agents can be administered orally and have a favorable safety profile compared to established anti-TNF agents.

Ayyar and Moss summarized the actual knowledge on extracellular vesicles including exosomes in intestinal inflammation. The compiled information illustrates that exosomes in particular and extracellular vesicles in general are capable of modulating gene expression and cellular functions, thereby critically impacting inflammation, immune responses, and composition of gut microbiota. As such these vesicles might be suitable biomarkers of IBD and molecular structures for therapeutic targeting specific cargos to the inflamed gastrointestinal tract.

Ferretti et al. provide an update on current sonographic methods to discriminate inflammation and fibrosis in Crohn’s disease, mainly focusing on studies investigating pathological features or the response to treatment as direct and indirect reference parameters. In their contribution, the authors highlight strengths and weaknesses of individual ultrasound methods and conclude that despite the promising results obtained with novel sonographic techniques such as contrast-enhanced ultrasound (CEUS) and sonoelastrography, several limitations must be addressed before using these techniques in routine clinical practice and trials.

Matsumoto et al. investigated potential therapeutic effects of the RXR agonist Net-3IB that was previously developed in their team in an experimental model of colitis induced through the adoptive transfer of CD45RB^high^CD4^+^ cells. The authors showed that Net-3IB ameliorates colitis by inhibiting both the expansion of Th1 cells and the activation of inflammatory macrophages locally in the colon. Thus, the small molecule inhibitor appears to be a promising candidate for the treatment of IBD.

Ke and colleagues addressed the question whether the anti-inflammatory effect of metformin in the intestine is due to a change of the microbiota. They examined this in the experimental colitis model of dextran sulfate sodium (DSS)-induced ulcerative colitis. Indeed the inflammation was attenuated by metformin. An increased expression of mucin 2 was noted. The gut microbiota changed under metformin by increasing protective *Lactobacillus* and *Akkermansia species*. With antibiotic exposure the anti-inflammatory and mucus-protecting effect of metformin was abolished. It is concluded that metformin-induced changes of the microbiota are therapeutically effective in ulcerative colitis.

The study by Nguyen et al. demonstrates the importance of the noncanonical NF-κB signaling pathway in IBD patients. In their study, the authors analyzed the expression of 88 target genes known to be associated with noncanonical NF-κB signaling in biopsy specimens that were collected during colonoscopy. Importantly, the expression of a number of genes was associated with increased gastrointestinal inflammation and in particular in patients unresponsive to anti-TNF agents. Although the study is based on a relatively small sample size, the study suggests that the noncanonical NF-κB signaling is an understudied pathway that critically impacts the pathogenesis of IBD.

All these 28 articles show that inflammation and fibrosis of the gastrointestinal tract and liver are complex problems. Currently, there are a number of real breakthroughs in this research area, but of course scientists and clinicians are still at the beginning in the identification of suitable therapeutic targets. Since the frequency of these malignancies and their complications currently increase dramatically, new strategies anti-inflammatory and anti-fibrotic therapies are urgently needed.

